# Automated algorithms for seizure forecast: a systematic review and meta-analysis

**DOI:** 10.1007/s00415-024-12655-z

**Published:** 2024-09-06

**Authors:** Ana Sofia Carmo, Mariana Abreu, Maria Fortuna Baptista, Miguel de Oliveira Carvalho, Ana Rita Peralta, Ana Fred, Carla Bentes, Hugo Plácido da Silva

**Affiliations:** 1 Department of Bioengineering, Instituto Superior Técnico, Universidade de Lisboa, Lisboa, Portugal; 2 Instituto de Telecomunicações, Lisboa, Portugal; 3 Neurophysiology Monitoring Unit EEG/Sleep Laboratory, Hospital de Santa Maria, Unidade Local de Saúde Santa Maria, Lisboa, Portugal; 4 Centro de Estudos Egas Moniz. Faculdade de Medicina da Universidade de Lisboa, Lisboa, Portugal; 5 LUMLIS The Lisbon ELLIS Unit | European Laboratory for Learning and Intelligent Systems, Lisboa, Portugal

**Keywords:** Automated seizure forecast, Seizure likelihood, mHealth, Seizures, Epilepsy, Systematic review

## Abstract

This study aims to review the proposed methodologies and reported performances of automated algorithms for seizure forecast. A systematic review was conducted on studies reported up to May 10, 2024. Four databases and registers were searched, and studies were included when they proposed an original algorithm for automatic human epileptic seizure forecast that was patient specific, based on intraindividual cyclic distribution of events and/or surrogate measures of the preictal state and provided an evaluation of the performance. Two meta-analyses were performed, one evaluating area under the ROC curve (AUC) and another Brier Skill Score (BSS). Eighteen studies met the eligibility criteria, totaling 43 included algorithms. A total of 419 patients participated in the studies, and 19442 seizures were reported across studies. Of the analyzed algorithms, 23 were eligible for the meta-analysis with AUC and 12 with BSS. The overall mean AUC was 0.71, which was similar between the studies that relied solely on surrogate measures of the preictal state, on cyclic distributions of events, and on a combination of these. BSS was also similar for the three types of input data, with an overall mean BSS of 0.13. This study provides a characterization of the state of the art in seizure forecast algorithms along with their performances, setting a benchmark for future developments. It identified a considerable lack of standardization across study design and evaluation, leading to the proposal of guidelines for the design of seizure forecast solutions.

**Supplementary Information:**

The online version contains supplementary material available at 10.1007/s00415-024-12655-z.

## Introduction

People with epilepsy (PWE), as well as their caregivers, live with the uncertainty of when the next seizure might happen, severely constraining some patients’ independence and entitlement to normalcy in day-to-day activities. Despite the seeming unpredictability of seizure occurrence, better-than-chance patient-based prediction of impending seizures has been reported [1, 2], which suggests the existence of preictal dynamics that may be leveraged by automated forecasting algorithms. In fact, PWE and caregivers have reported several non-physiological factors that, from preliminary observation, appear to be linked to seizure occurrence [3]. These include potential seizure triggers (such as alterations in sleep patterns [4]), the environment (such as changes in atmospheric pressure [5]), and cyclicity in seizures [6], which could be used as inputs for a forecast tool [3]. There have also been reports of physiological manifestations that precede seizures [7], including in electrodermal activity (EDA) [8] and electroencephalography (EEG) [9], as well as cyclic patterns in these (and other) biosignals, which appear to be phase locked with seizure occurrence [6, 10]. As such, forecasting of seizure likelihood has been the focus of a joint effort within the epilepsy community since the Epilepsy Foundation recognized its relevance in 2016 [11].

Some authors have implied that the forecast of seizures should be based on dynamics observed hours or days before the seizure occurs [12], including physiological alterations in signals like the EEG, EDA, and others, hereinafter called surrogate measures of the preictal state (SMPS). Others authors have gone further and restricted the basis of the forecast to intraindividual cyclic distribution of events (CDE) [7], which comprise cyclicity in the occurrence of seizures or even in certain physiological events, such as interictal epileptiform activity (IEA). Regardless, there is hardly any clear definition of what constitutes seizure forecast, and particularly what distinguishes this practice from seizure prediction. Instead, the major effort from the community toward this distinction has been on shifting the focus from a deterministic perspective (i.e., to raise, or not to raise, an alarm for an impending seizure) to assessing the body states that suggest a higher likelihood of seizure [12, 13].

In the last few years, several automated algorithms that attempt to gauge the likelihood of seizure occurrence in a highly individualized manner have been proposed in literature, and the reported performances are promising. However, no standardized review of the algorithms proposed has been attempted so far.

Moreover, the shift in perspective and problem statement (i.e., from a deterministic problem into a measure of likelihood) imposes additional challenges when it comes to evaluating and comparing forecast performances. Due to its inherent probabilistic nature, we may encounter situations in which the brain enters a state of high seizure likelihood without this actually translating into the occurrence of a seizure event [14, 15], challenging the traditional approaches to performance evaluation. Much like the task of seizure prediction lacked performance standards at the start of the 2000s [16], so does seizure forecast now.

In this paper, we review reported performance of automated algorithms that attempt to provide an individual’s likelihood of having a seizure within a given time window, based on intraindividual cyclic distribution of events and/or surrogate measures of the preictal state. This includes methods which aim to forecast seizure likelihood and that leverage seizure triggers, the environment, cyclicity in seizures, patterns in physiological data, or a combination of these. This review will address the following research questions:

- RQ1: What is the current state of automated seizure forecast?
- RQ2: Which data are the most relevant for the forecast of seizure likelihood?
- RQ3: Which approaches and metrics are most often used to assess forecast performance?

## Materials and methods

This review was performed in accordance with the Preferred Reporting Items for Systematic Reviews and Meta-Analyses (PRISMA) guidelines [17]. Details of the protocol for this systematic review were registered on International Prospective Register of Systematic Reviews (PROSPERO), under PROSPERO ID CRD42023478920.

### Concept definition

#### Forecast horizon

**Fig. 1:**
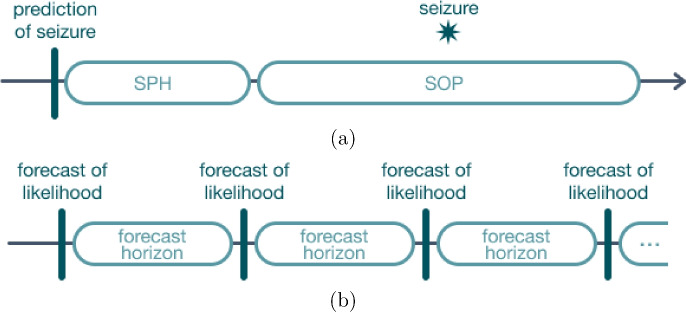
Illustration of the concepts of (**a**) SPH and SOP, where a correct prediction corresponds to an onset that occurs after SPH and within SOP; and (**b**) forecast horizon, where the forecasts are equally spaced in time in contrast with the task of seizure prediction

Seizure prediction and forecasting are often (incorrectly) used interchangeably. In seizure prediction, the main objective is to raise an alarm prior to a seizure occurring, providing the patient (or caregiver) the opportunity to act in accordance, either by taking fast-acting anti-seizure medication (ASM) or adopting protective measures. When developing seizure prediction algorithms, the concepts of seizure prediction horizon (SPH) and seizure occurrence period (SOP) arise. The corresponding definitions were initially proposed by Maiwald et al. [18] (as illustrated in Fig. 1a) and have since been adopted in several papers addressing seizure prediction and sometimes extended to forecasting [19–21]. SPH is defined as the period of anticipation of a seizure event, i.e., the time interval in which a seizure should not (yet) occur after an alarm is raised, while SOP is the interval of time (after SPH) within which the seizure onset is expected to arise.

Seizure forecast, on the other hand, attempts to provide the patient (or caregiver) with an indicator of seizure likelihood for a specific time period after the forecast, called the forecast horizon (Fig. 1b). This concept denotes the time interval in the future for which the forecast is generated [22]. As such, the forecast horizon is not a direct counterpart to the concepts of SPH and SOP.

#### Retrospective vs pseudo-prospective approach

The train–test approach consists of the methodology used for model evaluation and can be either retrospective or (pseudo-)prospective.

In a retrospective approach, the data is randomly divided into training and test sets without considering the temporal relationship of the data points.

Conversely, in a prospective approach, the model is trained only on past data and evaluated in real time on future data. Similarly, in a pseudo-prospective approach, the prospective evaluation is emulated by using retrospective data, but dividing it into training and test sets so that the model is trained only on past data and evaluated in data posterior to that, thus also respecting time dependencies.

#### Deterministic vs probabilistic forecast

In seizure forecast, we encounter a view that challenges the traditional approaches to performance evaluation. Most commonly, when assessing the performance of a prediction of seizure, the concepts of SPH and SOP are leveraged, where a correct prediction corresponds to an onset that occurs after SPH and within SOP. However, when working with forecasts, instead of having an alarm raised, a likelihood is received at equally spaced intervals of time.

To handle this issue, some studies opt for converting the forecasts into binary high/low risks (using discriminative thresholds), thus allowing the use of traditional (i.e., deterministic) performance measures. A number of papers adopt metrics such as sensitivity (Sen), accuracy (Acc), and area under the ROC curve (AUC). However, these methodologies are highly dependent on the chosen SPH and SOP. In fact, by replacing the categorical notion of seizure occurrence (i.e., it either occurred when expected, or it did not) with a likelihood, we may encounter situations in which the brain enters a state of high seizure likelihood without this actually translating into the occurrence of a seizure event [15, 23]. Therefore, the traditional measures of performance are usually replaced or complemented with alternatives that allow to properly assess the clinical utility of the forecasts, such as Brier Score (BS) and Brier Skill Score (BSS).

### Systematic review

A systematic literature search was carried out on the databases IEEE Xplore, Scopus, and PubMed, as well as on the Web of Science Core Collection. The search was conducted using the query ((automated OR automatic OR algorithm OR machine learning OR deep learning OR artificial intelligence) AND (forecast OR risk OR likelihood OR prediction OR cyclic* OR rhythm*) AND (epilepsy OR seizure)) on the title, abstract, and keywords of the studies. A first search was conducted up to November 2, 2023. Another search was conducted on May 10, 2024.

From the identified records, built-in automation tools of the search engines were used to filter the results according to language (resulting in the exclusion of all records that were not written in English, Portuguese, Spanish, or French), as well as according to document type (resulting in the exclusion of all reviews). Then, the corresponding references were uploaded into the Rayyan software,^1^ in which duplicates were automatically identified. Duplicates were resolved by the Rayyan built-in automation tool for the cases where similarity was equal to or larger than 90%; the remaining duplicates were resolved manually by one of the reviewers (A.S.C.). The titles and abstracts were screened by three independent reviewers (A.S.C., M.F.B. and M.O.C.), resulting in the exclusion of the records that were not considered relevant. Conflicts were resolved in an open discussion between all reviewers. The inclusion criteria were:

1. Proposal of an original real-time forecast algorithm for automatic human epileptic seizure likelihood assessment, that is patient-specific, based on intraindividual cyclic distribution of events and/or cyclic distribution of events.
2. Any cohort dimension.
3. Records written in English, Portuguese, Spanish, or French.
4. Published as an article, proceeding paper, or abstract.

After screening, the full-text documents were retrieved and assessed for eligibility by two independent reviewers (A.S.C and M.A.). Non-trivial cases were decided in an open discussion between the two reviewers. Studies were excluded if:

1. Separate sets of data were not used for training and testing of the forecast algorithm.
2. The proposed algorithm did not provide a measure of seizure likelihood/risk.
3. Failed to report at least one of the following data: methodology proposed for forecast of seizure likelihood; data considered for the algorithm; forecasting horizon; methodology and results on the evaluation of seizure forecast performance.

Finally, A.S.C extracted the data, which included:

1. Input data: source, type, description.
2. Cohort: dimension, median/total duration of recording, median/total number of seizures.
3. Methodology: type of algorithmic approach, forecast horizon, train–test approach.
4. Results: metrics used to evaluate performance, reported performance.

### Meta-analysis

A meta-analysis was performed to quantify the current state of seizure forecast. Studies were stratified according to the type of input data given to the algorithms, which can be categorized as SMPS, CDE, or a combination of these, following the distinction provided in [7].

The two most highly reported deterministic and probabilistic metrics were chosen for the meta-analysis. As such, random-effects meta-analysis with the restricted maximum likelihood (REML) method was used to obtain pooled estimates of AUC and BSS. The AUC, which ranges from 0 to 1 (perfect score), can measure the forecast performance based either on true positive rate versus false positive rate or sensitivity versus portion of time in false warning. It is often used in seizure prediction literature as the main performance outcome, including in previous seizure prediction/forecast challenges [24, 25]. BSS, on the other hand, is a probability score that provides a measure of improvement over a naive forecast, ranging from -$\inf$ to 1, where 0 corresponds to a performance equivalent to the naive forecast.

A weighting scheme was also employed, where the variance of each study was weighted by its sample size. This approach ensured that studies with larger sample sizes contributed more to the overall analysis.

Finally, sources of heterogeneity were also explored through subgroup analysis for each potential moderator variable. For the eligible cases^2^ the moderator effect of the variables on heterogeneity was quantified by including them as covariates in the meta-analysis model, and evaluated according to changes in $\hbox {I}^2$. Five sources of heterogeneity were considered apart from the type of input data: source of data (i.e., dataset used), input data (EEG vs heart rate vs seizure times vs other inputs), forecast horizon (<1 h, 1 h, 24 h), train/test approach (retrospective vs prospective/pseudo-prospective), and study (i.e., algorithms proposed by the same study).

Statistical analysis was performed using the metafor R library and, when applicable, statistical significance was set to 0.05.

## Results

**Fig. 2:**
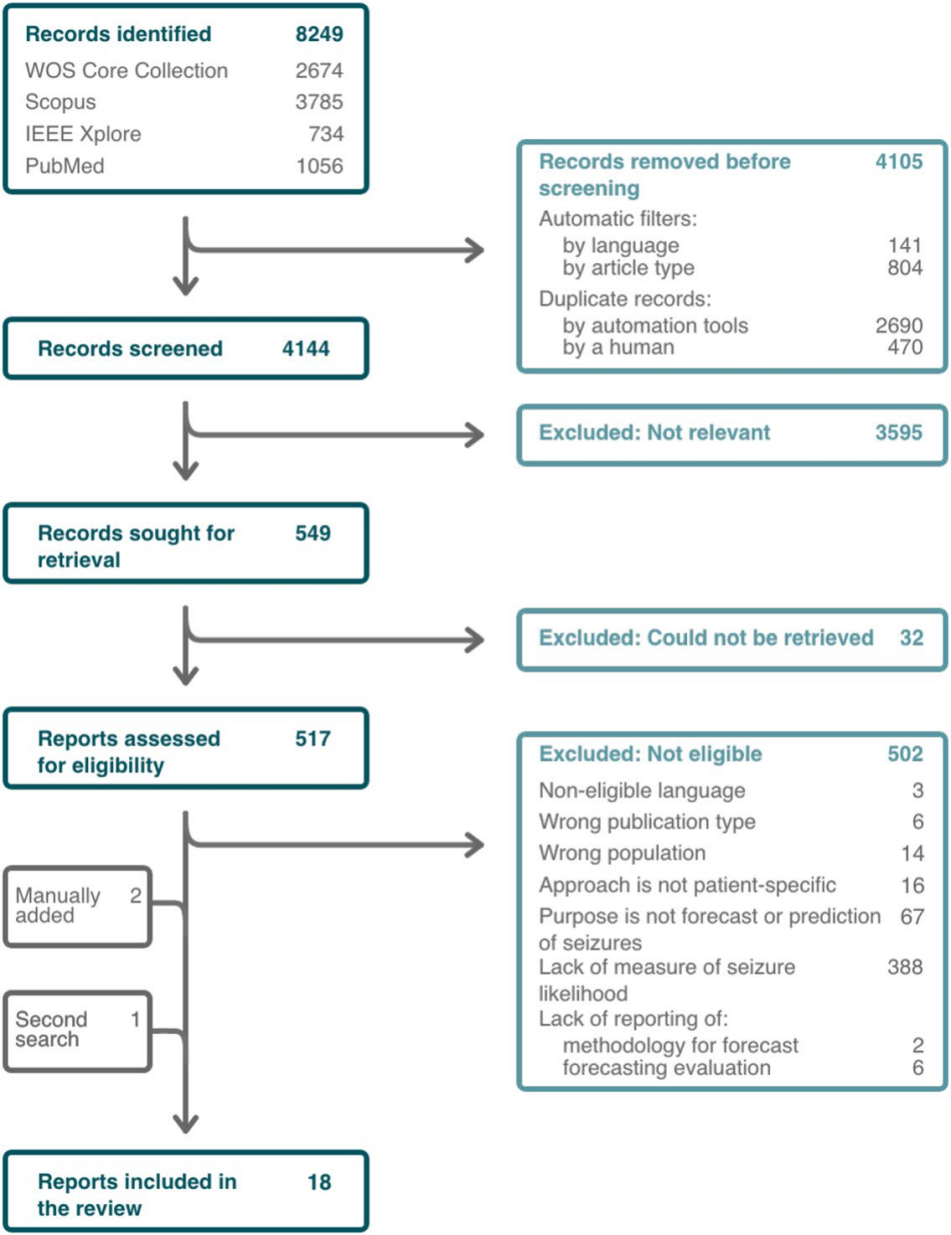
PRISMA flow diagram depicting the number of records identified, included and excluded, as well as the reasons for exclusion. Four databases and registers were searched, resulting in 15 included reports, with 3 additional reports being included at a later stage. A total of 18 reports were included in the review

A total of 8249 records were identified, from which 945 were removed according to publication type and language (see Fig. 2 for the PRISMA flow diagram). Additionally, 3160 duplicates were removed. Of the remaining, 4144 records were screened for relevance to the review topic and 549 of these were sought for retrieval of the full records. Out of the 549 records, 32 could not be retrieved and a further 502 studies were excluded for not meeting at least one of the eligibility criteria. Finally, three additional reports were included, one as a result of the second search and the other two were manually added. In total, 18 studies were included in the systematic review.

### Study characteristics

The results for the 18 studies are summarized in Tab. 1. A total of 419 patients participated in the studies (median of 12, interquartile range (IQR) of 8.25$-$17.25), and a total number of 19442 seizures were reported across studies^3^. The duration of the total/recorded data varied greatly across studies, and even within the same sample, with some studies encompassing less than 1 week [28] and others up to 9 years [22].

**Table 1:** Summary of characteristics of the studies included in the systematic review

First author, year	Data source	Sample size	Duration in days (median)	# Seizures (median)	Type of input data	Algorithmic approach	Reported metrics
Attia, 2021 [33]	24/7 EEG SubQ trial	1	230	22	SMPS	LSTM	AUC, Sen, % Sig., FPR
Chen, 2022 [34]	NeuroVista trial	15	557	151	CDE	Phase modeling	AUC, Sen, TiW, % Sig., GSS
Cook, 2013 [29]	NeuroVista trial	11	265	31	SMPS	k-NN/decision tree type classifier	Sen, TiW, Likelihood ratio
Costa, 2024 [35]	EPILEPSIAE database	40	5	5	SMPS	SVM, LR, SNN	BSS, Sen, TiW, FPR, BS
Cousyn, 2022 [36]	Self-collected	10	Mean: 10.7	2	SMPS	SVM	AUC, Acc, F1-score, BSS, BS
Cousyn, 2023 [28]	Self-collected	15	11	Mean: 25	SMPS	SVM	AUC, Acc, Sen, Spe, F1-score, BS
Karoly, 2017 [37]	NeuroVista trial	9	459	102	SMPS; CDE; both	LR; phase modeling; ensemble through LR weight updating	AUC, BSS, Sen, TiW
Karoly, 2020 [38]	Self-collected and NeuroVista trial	50	Mean: 336	Mean: 109	CDE	Phase modeling	AUC, Acc, TiW, % Sig.
Leguia, 2022 [27]	NeuroPace trial and 24/7 EEG SubQ trial	161	1722; 85	143	SMPS; CDE	GLM	AUC, BSS, % Sig.
Maturana, 2020 [39]	NeuroVista trial	14	512	151	CDE	Phase modeling	Sen, TiW
Nasseri, 2021 [40]	Self-collected	6	242	16	SMPS	LSTM	AUC, Sen, TiW, % Sig
Payne, 2020 [20]	NeuroVista trial	8	Mean: 548	157	CDE	Phase modeling; ensemble through naive Bayes	AUC, % Sig.
Proix, 2021 [22]	NeuroPace trial	18	Mean: 1484	Mean: 43	SMPS; CDE; both	GLM	AUC, BSS, % Sig.
Stirling, 2021 [41]	self-collected	11	435	94	both	LSTM + RF (ensemble through LR)	AUC, TiW, % Sig., BS
Stirling, 2021 [42]	Minder sub-scalp system trial	1	183	134	CDE	RF + LR (ensemble through sequential input)	AUC, Sen, TiW
Truong, 2021 [21]	EPILEPSIAE database	30	4	Mean: 9	SMPS; both	Bayesian CNN	AUC
Viana, 2022 [43]	24/7 EEG SubQ trial	6	80	17	SMPS	LSTM	AUC, Sen, TiW, % Sig., FPR
Xiong, 2023 [44]	self-collected	13	495	71	CDE	Phase modeling	AUC, BSS, Sen, TiW, % Sig

Duration and seizure number are given as median, unless stated otherwise. SMPS: surrogate measures of the preictal state; CDE: cyclic distribution of events. CNN: convolutional neural network; GLM: generalized linear model; k-NN: k-nearest neighbors; LR: logistic regression; LSTM: long short-term memory network; SNN: shallow neural network; RF: random forest; SVM: support vector machine. AUC: area under the ROC curve; BS: Brier Score; BSS: Brier Skill Score; FPR: false positive rate; GSS: geometric mean of sensitivity and specificity; Sen: sensitivity; Spe: specificity % Sig: percentage of patients with significant forecasts; TiW: time in warning

There was little consistency regarding the source of data used across the body of studies, with 5 studies relying on self-collected data when evaluating their proposed algorithms and the remaining 12 using previously documented datasets. The NeuroVista trial [29] was the most commonly reported (6, one of them in combination with self-collected data), consisting of 15 patients with focal seizures submitted to ambulatory intracranial EEG monitoring for more than 80 days. Two studies reported using data from the NeuroPace trial [30], where an implanted brain-responsive neurostimulator was validated in patients with disabling partial or generalized tonic–clonic seizures, for a mean follow-up period of 5.4 years. Data from the 24/7 EEG SubQ trial [31] was used in three studies (one of them in combination with data from the NeuroPace trial), consisting of subcutaneous EEG recordings of nine participants with temporal lobe epilepsy, for up to 3 months. Two studies relied on data from the EPILEPSIAE database [32], which was created under a joint European project and is the largest collection of hospital EEG recordings, with data from more than 250 patients. Finally, a single study used data from the Minder sub-scalp system trial (ACTRN 12619001587190), where six patients were implanted with a sub-scalp EEG monitoring system. Moreover, the majority (14/18) used data collected with mobile technology, which included subcutaneous and intracranial EEG implants, smartwatches and other wrist-based devices, as well as mobile applications.

**Fig. 3:**
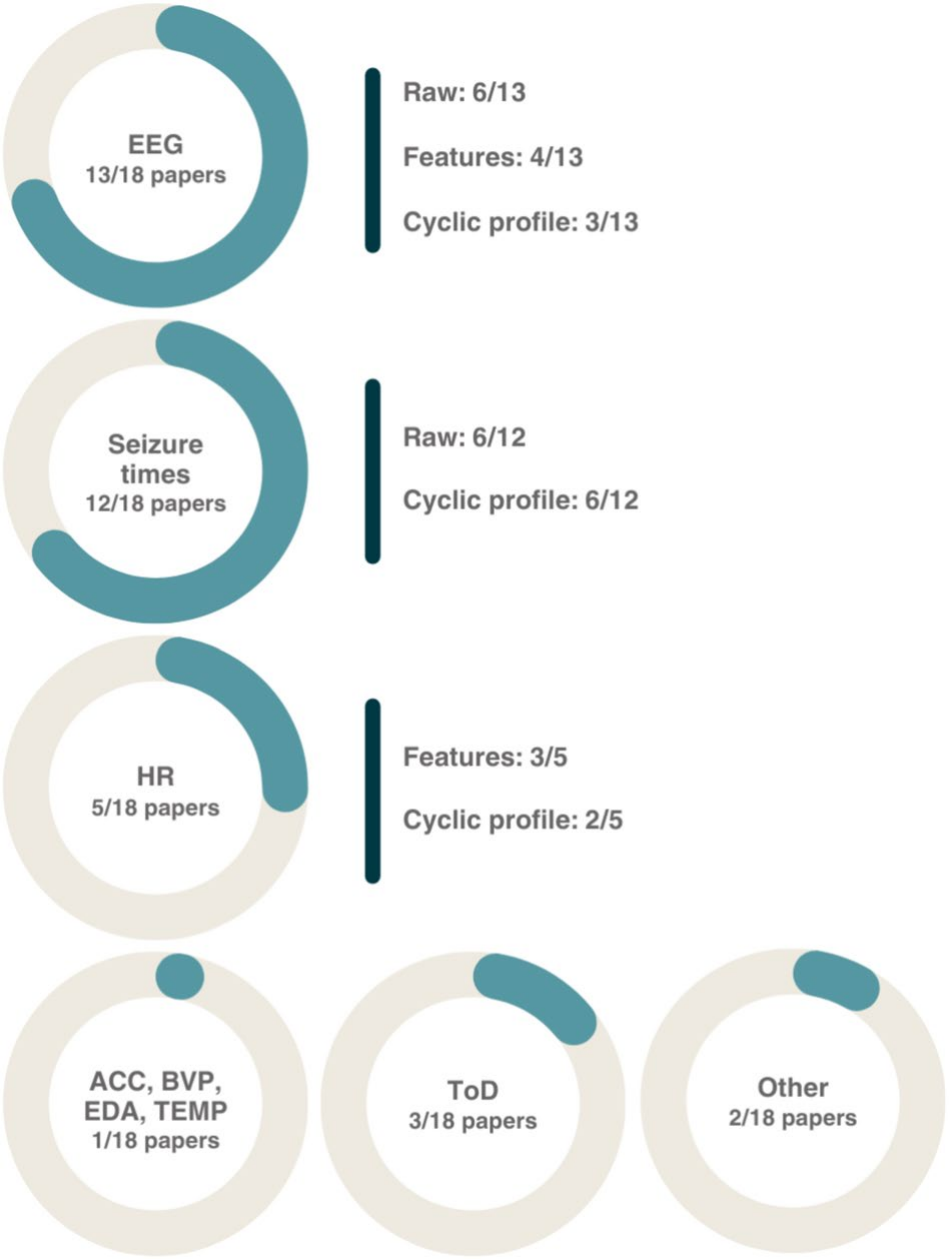
Summary of reported input data across the 18 papers included in the review, along with how the data were given to the algorithms, when applicable (i.e., raw vs features vs cyclic profile). *ACC* accelerometry, *BVP* blood volume pulse, *EDA* electrodermal activity, *EEG* electroencephalography, *HR* heart rate, *TEMP* temperature, *ToD* time of day, *Other* sleep, activity, weather

Regarding input data, seven studies used only SMPS, six reported using solely CDE, four proposed algorithms that use both types of input data, and one study proposed algorithms using both types separately. Figure 3 depicts the use of the different data used as input in the 18 papers, as well as how the data was given to the algorithmic approaches, when applicable. EEG was the most widely used physiological signal (13/18), either as a raw input (5/13), through features derived from it (4/13), or through cyclic patterns extracted from those features (3/13). Photoplethysmography (PPG) and electrocardiography (ECG) followed as sources of heart rate (HR) (5/18). Similarly to EEG, HR was not only explored as a surrogate measure of the preictal state, but also as a source of CDE (2/5). Apart from the EEG, seizure times were the most commonly used input data, being used in 12 out of the 18 studies, and equally explored as a raw input or through cyclic profiles derived from them. Other, less commonly reported data inputs included other peripheral physiological signals, the time of day (ToD), and behavioral/external conditions.

Finally, despite the probabilistic framework of the task of seizure forecast, less than half the studies (8/18) reported probabilistic evaluation metrics, while all studies reported deterministic ones. AUC was the most reported metric (15/18), followed by Sen and time in warning (TiW) (11/18), and % of patients with significant forecasts (10/18). BS and BSS were reported in eight and six studies, respectively^4^. Moreover, when using both types of train–test approaches, i.e., retrospective vs (pseudo-)prospective, most studies (5/7) reported a different set of metrics for each.

### Descriptive analysis

Table 2 summarizes the characteristics of all considered algorithms. Some studies comprise multiple table entries, corresponding to algorithms whose characteristics differ significantly with regard to the following attributes: input data, forecast horizon, and train–test approach. For each of these unique characteristics’ configurations, only the best performance is reported. AUC and BSS scores are presented, whenever reported in the studies. An extension of this table, including all reported performance metrics, is available in Supplementary file 1.

**Table 2:** Summary of characteristics of the studies included in the systematic review

First author, year	Sample size	Total # seizures	Type of input data	Input data	Horizon	Approach	AUC	TiW	% Sig	BSS	BS
Attia, 2021 [33]	1	15	SMPS	EEG	1 h	R	0.75		100%		
				EEG, ToD			0.81		100%		
Chen, 2022 [34]	15	2398	CDE	EEG cyclic profile	5min	R	0.91±0.08	L:80%	100%		
	12	1976				P	0.75±0.10	L:80%	92%		
Cook, 2013 [29]	11	154	SMPS	EEG features	5min	R		H:27.8% L:42.2%			
	10	323				P		H:23% L:45%			
Costa, 2024 [35]	40	224	SMPS	EEG	10min	P		H:10%		0.01±0.15	0.18±0.1
Cousyn, 2022 [36]	10	38	SMPS	EEG features	24 h	R	0.79				
						P				0.72±0.22	0.13±0.11
Cousyn, 2023 [28]	15	47	SMPS	HR features	24 h	R	0.79±0.17				
	14					P					0.3 [0.18;0.48]
Karoly, 2017 [37]	9	1383	SMPS	EEG features	30min	R	0.79±0.08				
						P		H:25.3%		0.05±0.06	
			CDE	Seizure cyclic profile				H:24.4%		0.05±0.03	
			Both	ensemble				H:24.7%		0.11±0.07	
Karoly, 2020 [38]	50	5450	CDE	Seizure cyclic profile	5min	P	0.85±0.05	H:14.8% L:67.1%	100%		
Leguia, 2022 [27]	161	(*)	SMPS	Seizure times	24 h	R	0.63±0.08		45%	0.08±0.10	
				EEG features			0.65±0.05		31%	0.08±0.08	
			CDE	EEG cyclic profile			0.69±0.06		60%	0.10±0.09	
Maturana, 2020 [39]	14	2871	CDE	EEG cyclic profile	2min	P	H:7.75%				
Nasseri, 2021 [40]	6	278	SMPS	ACC, BVP, EDA, TEMP, HR, ToD, signal quality metrics	1 h	R	0.75±0.15	H:5 (h/day)	83%		
Payne, 2020 [20]	8	1236	CDE	Sleep features cyclic profile	10min	P	0.63±0.11		62%		
				Weather features cyclic profile			0.55±0.10		62%		
				Seizure cyclic profile			0.69±0.08		75%		
				ensemble			0.68±0.11		75%		
Proix, 2021 [22]	18	767	SMPS	EEG features, seizure times	24 h	P	0.61 [0.59,0.64]		11%	0.02 [0.01,0.04]	
					1 h		0.60 [0.57,0.65]		67%	0.01 [0.00,0.01]	
			CDE	Seizure and EEG cyclic profiles	24 h		0.73 [0.65,0.76]		83%	0.17 [0.11,0.26]	
					1 h		0.70 [0.64,0.77]		94%	0.02 [0.01,0.02]	
			Both	ensemble	1 h		0.75 [0.69,0.81]		100%	0.03 [0.02,0.06]	
Stirling, 2021 [41]	11	1493	Both	HR features, sleep features, activity features, HR cyclic profile, seizure times	24 h	R	0.66±0.11	median L:18%	91%		21.91±0.72
	8	1078				P	0.59±0.16		50%		
	11	1493			1 h	R	0.74±0.10	median H:14%	100%		0.17±0.05
	8	1078				P	0.65±0.18		88%		
Stirling, 2021 [42]	1	134	CDE	Seizure and EEG cyclic profiles	1 h	P	0.88	H:26% L:63%			
Truong, 2021 [21]	30	261	SMPS	EEG	30min	R	0.69				
			Both	EEG and seizure cyclic profile			0.69				
Viana, 2022 [43]	6	103	SMPS	EEG	1 h	R	0.65±0.17	H:33.3%	67%		
				EEG, ToD			0.73±0.07	H:31.47%	83%		
Xiong, 2023 [44]	13	2247	CDE	Seizure and HR cyclic profiles	24 h	R	0.70±0.15		85%	0.20±0.23	
	6	2514				P	0.74±0.17		83%	0.17±0.17	
	13	2247			1 h	R	0.71±0.12	H:27%	69%	0.04±0.03	
	6	2514				P	0.76±0.07	H:18%	67%	0.05±0.05	

Duration and seizure number are given as median, unless stated otherwise. *SMPS* Surrogate measures of the preictal state, *CDE* cyclic distribution of events, *CNN* convolutional neural network, *GLM* generalized linear model; *k-NN* k-nearest neighbors; *LR* logistic regression, *LSTM* long short-term memory network, *SNN* shallow neural network, *RF* random forest, *SVM* support vector machine, *AUC* area under the ROC curve, *BS* Brier Score, *BSS* Brier Skill Score, *FPR* false positive rate, *GSS* geometric mean of sensitivity and specificity, *Sen* sensitivity, *Spe* specificity, % *Sig* percentage of patients with significant forecasts, *TiW* time in warning

**Fig. 4:**
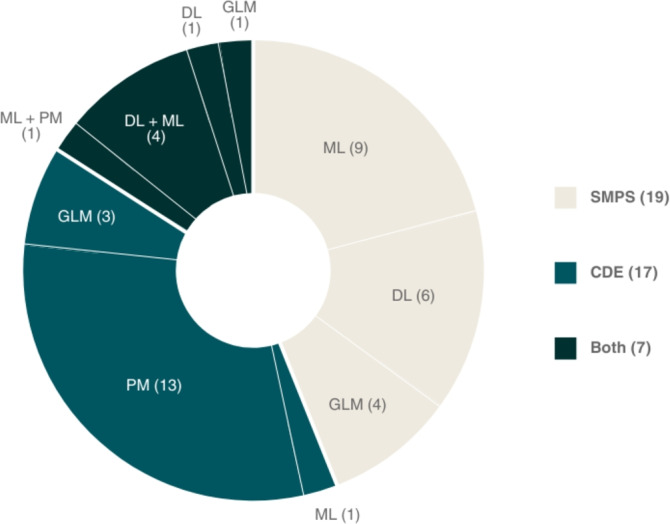
Summary of types of input data reported across the 43 algorithms, along with the category of algorithmic approach used. *SMPS* Surrogate leasures of the preictal state, *CDE* cyclic distribution of events, *ML* machine learning, *DL* deep learning, *GLM* Generalized linear lodel, *PM* phase modeling

The 18 studies proposed a total of 43 algorithms with unique configurations. The most common type of input data was SMPS (19/43), which included the time series of the physiological signals, features extracted from them, times when seizures occurred, and ToD. Seventeen algorithms relied on CDE, which were either based on times of seizure occurrence, IEA, HR, sleep, or weather features. The remaining algorithms (7/43) used a combination of the previous inputs, as well as features extracted from patient’s activity. Figure 4 provides a summary of the types of input data reported across the 43 algorithms, along with the category of algorithmic approach used.

Remarkably, most algorithms relied on methodologies for phase modeling when leveraging CDE (13/17 and 1/7), which implies the identification of significant cycles or the use of histograms of the times of previous events. The remaining opted for the use of generalized linear models (GLMs) (3/17 and 1/7), deep learning (DL) models such as convolutional neural networks (CNNs) (1/7), or more classic machine Learning (ML) models such as random forest (RF) and logistic regression (LR) (1/17 and 4/7). On the other hand, algorithms using SMPS relied most commonly on classic ML algorithms (9/19 and 5/7), including support vector machines (SVMs) and LR, while the remaining opted for GLMs (4/19 and 1/7), or long short-term memory networks (LSTMs) and CNNs (6/19 and 5/7).

It was not uncommon for studies to evaluate their algorithms through a dual approach of retrospective and (pseudo-)prospective train–test schemes (7/18). In such cases, the retrospective period was used for development of the algorithm, often in a cross-validation scheme, providing an estimate of algorithm performance. Then, the (pseudo-)prospective period was used to simulate a real-world scenario, often being iteratively updated with the “new” data, in which the algorithm was validated in a setting analogous to the one it was designed to function.

### Meta-analysis

**Fig. 5:**
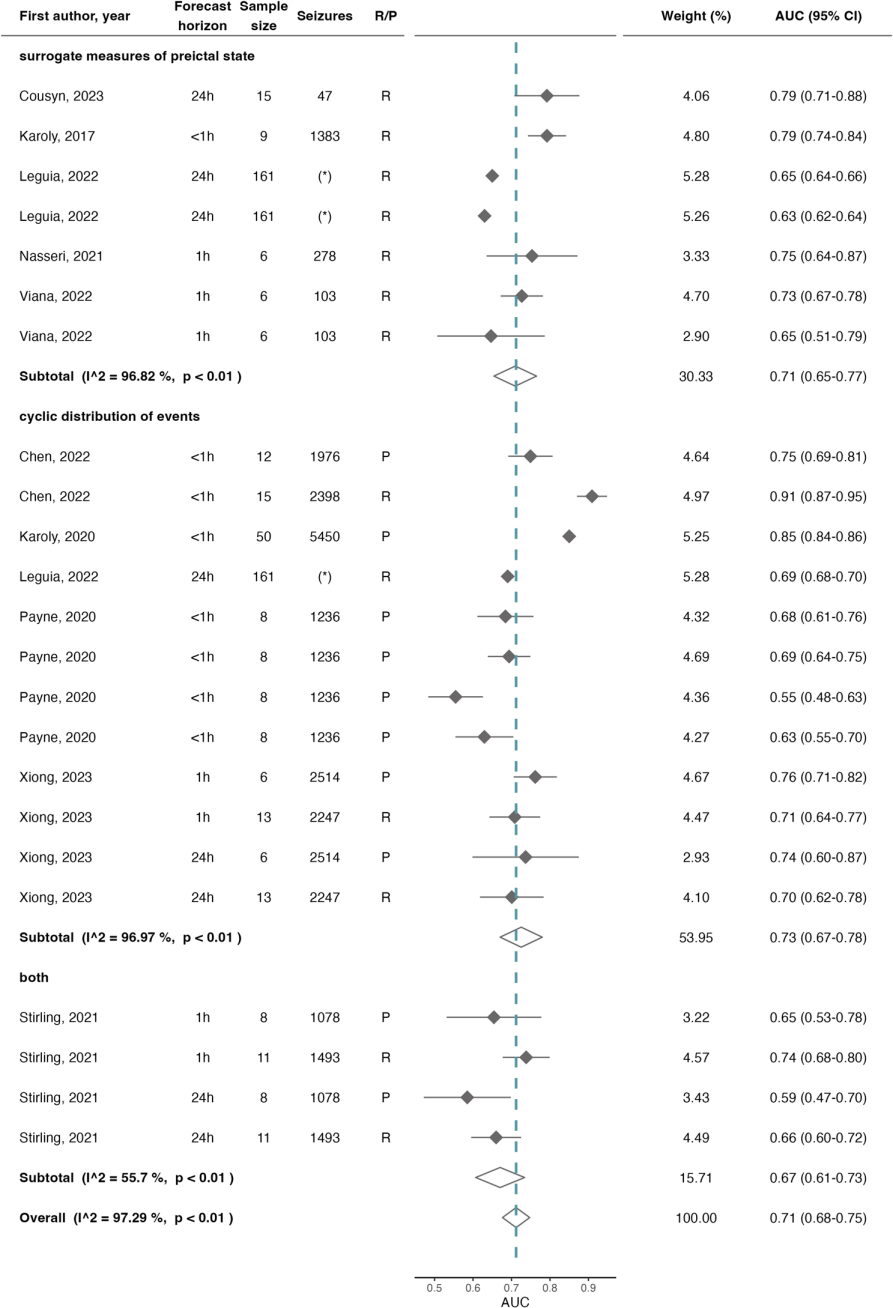
Forest plot of AUC of seizure forecast algorithms, overall and stratified by the type of input data given to the algorithms. The dashed line illustrates the estimated overall AUC and the diamonds represent either the overall or subgroup summary. Additional information provided includes the horizon of the forecast, the sample size, total number of seizures, and the train–test approach (which was either (pseudo-)prospective, P, or retrospective, R). *AUC* area under the ROC curve. (*) Identifies the algorithm proposed by [27], since they only reported the median number of seizures (143, IQR of 13-1233)

**Fig. 6:**
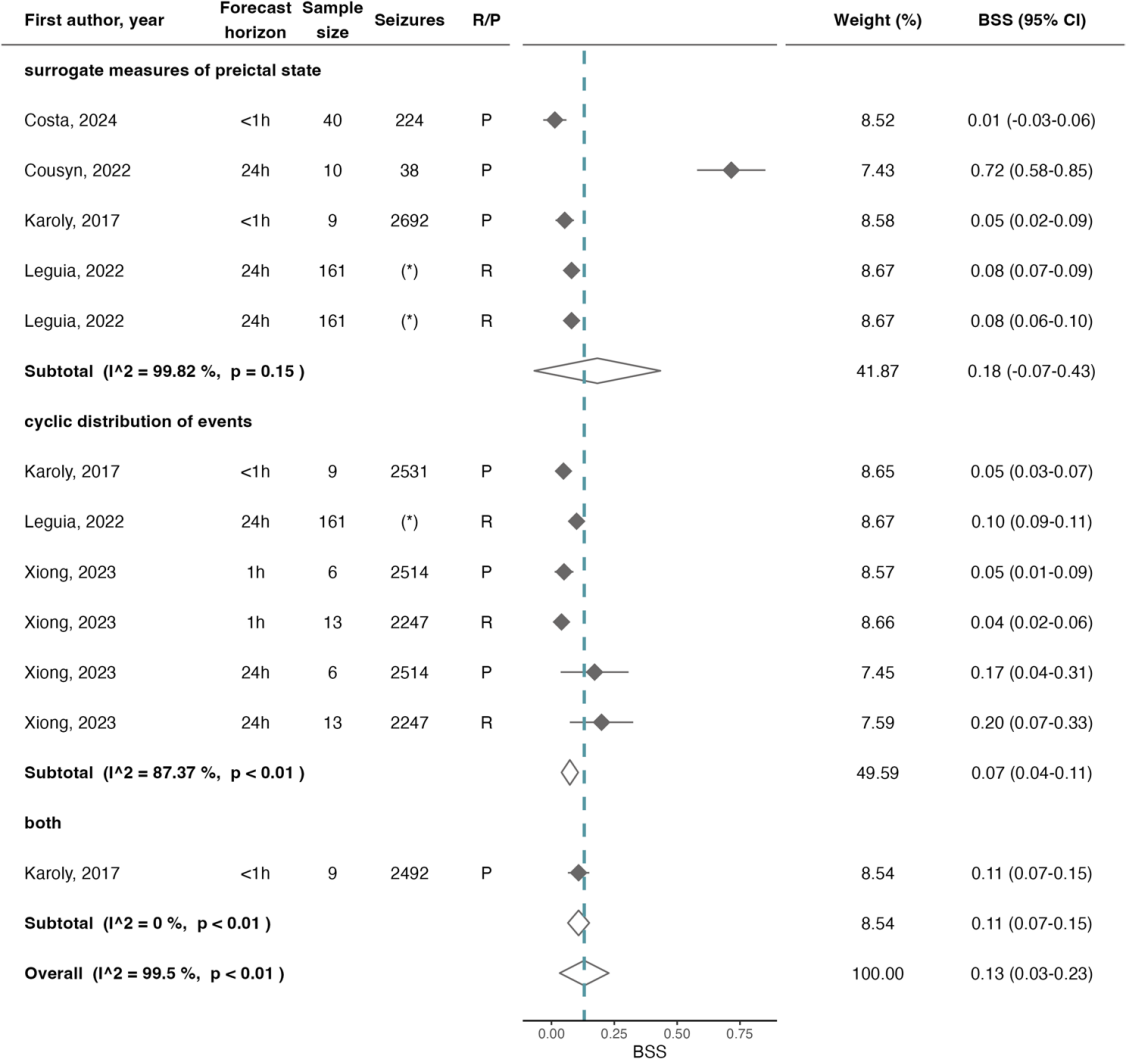
Forest plot of BSS of seizure forecast algorithms, overall and stratified by the type of input data given to the algorithms. The dashed line illustrates the estimated overall BSS, and the diamonds represent either the overall or subgroup summary. Additional information provided includes the horizon of the forecast, the sample size, total number of seizures, and the train–test approach (which was either (pseudo-)prospective, P, or retrospective, R). *BSS* Brier Skill Score. (*) Identifies the algorithm proposed by [27], since they only reported the median number of seizures (143, IQR of 13–1233)

Out of the 43 entries, 23 were eligible for the meta-analysis with AUC and 12 with BSS.^5^ The results of the meta-analysis are summarized in Fig. 5 and Fig. 6 for AUC and BSS, respectively. The overall mean AUC was 0.71 (95% confidence interval (CI)=0.68$-$0.75, $\hbox {I}^2$=97.29%), which was further stratified according to the type of input data given to the algorithms. The subtotal AUC for algorithms using only SMPS was 0.71 (CI=0.65$-$0.77, $\hbox {I}^2$=96.82%), for algorithms relying solely on CDE was 0.73 (CI=0.67$-$0.78, $\hbox {I}^2$=96.97%), and for algorithms that used both types of input data was 0.67 (CI=0.61$-$0.73, $\hbox {I}^2$=55.70%).

The overall mean BSS was 0.13 (CI=0.03$-$0.23, $\hbox {I}^2$=99.50%). The subtotal BSS for algorithms using only SMPS was 0.18 (CI=$-$0.07$-$0.43, $\hbox {I}^2$=99.82%) and for algorithms relying solely on CDE was 0.07 (CI=0.04$-$0.11, $\hbox {I}^2$=87.37%). A single algorithm using both types of input data reported the BSS score at 0.11 (CI=0.07$-$0.15).

When considering the five potential sources of heterogeneity (source of data, input data, forecast horizon, train/test approach, and study), only forecast horizon for AUC and train/test approach for AUC and BSS were eligible for quantification of moderator effect. However, none provided a significant explanation on heterogeneity, accounting for less than 1% change in $\hbox {I}^2$.

Regarding subgroup analysis of the remaining variables, some insights may be ascertained (all forest plots illustrating the subgroup analysis are provided as Supplementary file 2). While decreases in heterogeneity were observed in a set of subgroups in the case of BSS for source of data and forecast horizon, these subgroups were for the most part composed of results from the same studies. In fact, subgrouping by study also revealed equivalent decreases in heterogeneity for those same studies. As such, in the case of BSS, it was not possible to infer which of the two variables was the moderator.

In the case of AUC, subgrouping by study also revealed significant decreases in heterogeneity, with several subgroups presenting small to moderate $\hbox {I}^2$ values. Surprisingly, decreases in heterogeneity were also observed in one subgroup of input data and another subgroup in forecast horizon, which were not explained by the study variable. The former corresponds to the algorithms that used seizure times in combination with other input data, and the latter to the approaches that output forecasts with a horizon of 1 h.

## Discussion

This systematic review and the meta-analyses provide a characterization of the state-of-the-art of seizure forecast algorithms along with their performances, setting a benchmark for future developments. It addresses the relevance of classic prediction performance, while also reiterating the need for human-interpretable measures of likelihood and accompanying performance metrics. Hence, two meta-analyses are provided, one on AUC (a deterministic performance metric) and another on BSS (a probabilistic performance metric).

### Overview of the proposed approaches and performance

This systematic review allowed for the characterization of the current state of automated seizure forecast, effectively addressing RQ1. Firstly, it revealed a considerable diversity of the proposed approaches in regard to study design, including factors such as type of input data, algorithmic approaches, methodology for training and testing, and forecast horizons. While most proposed approaches relied on SMPS, an almost equal amount opted for exploring CDE. Moreover, ML and DL remain the most common type of algorithmic approach when SMPS is used as an input, however, phase modelling was highly favored for CDE.

The meta-analysis with the studies reporting AUC revealed an overall mean score of 0.71 (CI=0.68$-$0.75, $\hbox {I}^2$=97.29%), with a large heterogeneity across studies. The subgroup analysis showed small to moderate heterogeneity values for algorithms that used seizure times in combination with other input data and for the approaches that proposed forecasts with a horizon of 1 h. This insight potentially indicates a more reliable effect estimate for these two subgroups, with AUC of 0.70 (CI=0.67$-$0.73, $\hbox {I}^2$=0.00%) and 0.73 (CI=0.70$-$0.76, $\hbox {I}^2$=35.52%), respectively. Given that forecasts are often considered excellent when the AUC is above 0.9, these results indicate a reasonable performance while also showing opportunity for improvement in regard to this evaluation metric.

Furthermore, the near-zero BSS (0.13, CI=0.03$-$0.23) demonstrates only a slight improvement in performance over a naive forecast. This result underscores the need for a larger focus on the development of algorithms that are optimized for accurate portrayals of the true likelihood of a seizure occurring, instead of relying on machine-trained, threshold-based interpretations of those likelihoods.

Addressing RQ3, despite the efforts of the epilepsy community towards shifting the focus from a deterministic perspective to assessing the body states that suggest a higher likelihood of seizure, a majority of studies still relied entirely on deterministic performance metrics to evaluate their algorithms, with less than half the studies reporting probabilistic metrics, namely BS and BSS. The most common deterministic metrics were AUC, Sen, and TiW, requiring the conversion of the measures of seizure likelihood (either numerical or categorical, i.e., risk levels) into a classification of preictal/interictal segments. This reversion back to the classic notion of seizure prediction hinders the use case of seizure forecast.

Interestingly, there was not a considerable difference in AUC or BSS performance across subgroups. While these results should be interpreted with caution, due to the substantial unexplained heterogeneity and the small number of studies in each subgroup, they suggest comparable performances from both types of input data. While EEG patterns have historically been the most explored source of data due to its inherent relation to seizure onset, the pooled AUC and BSS performances along with the heterogeneity analysis obtained in this meta-analysis would suggest that other sources of data, including the timings of seizure occurrence, peripheral physiological data, or even sleep patterns may hold relevant insights into the non-deterministic nature of seizure occurrence. However, given the observed heterogeneity, at this stage it is not possible to address RQ2 and provide a directional statement in regard to which data are the most relevant for the forecast of seizure likelihood.

#### Guidelines for seizure forecast

This systematic review revealed a severe lack of standardization across sources of data (i.e., datasets), train–test approaches (i.e., retrospective vs pseudo-prospective), forecast horizons (i.e., daily, hourly, or on the order of minutes), evaluation methodologies (i.e., segment based vs event based, as described in [45]), and the metrics of performance (i.e., deterministic vs probabilistic). In fact, the lack of standardization in the literature is one of the major bottlenecks identified by this review. While the objective of this work is not to establish which are the best practices or methods, future developments may propose new approaches that are comparable to the studies included in this review with respect to three domains:

1. Data source: It is evidently desirable to benchmark any new approach in publicly available datasets, since it allows for direct comparison of results. However, given the novelty of this area of research, publicly available datasets may not include all input data that the readers intend to explore. For this reason, self-collected datasets may offer significant added value and should, whenever possible, be made publicly available.
2. Analysis: While there is no directional insight regarding the preferred value for horizon of the forecasts, several studies have reported community preferences regarding this design factor [46–48] which can guide future developments. Moreover, novel approaches towards seizure forecast should explicitly state this variable, as it is crucial to not only make them comparable to the literature, but also to inform the epilepsy community how that approach would be applied as a real-world tool. Furthermore, as distinct train/test methodologies preclude quantitative comparison between two proposed approaches, we advise future developments to evaluate algorithms in a (pseudo-)prospective manner, as it provides a more comprehensive estimate on the algorithms’ capacity to generalize [45] in comparison to retrospective evaluation. However, purely prospective studies may not be feasible due to resource constraints. In such cases, pseudo-prospective approaches, while not accounting for implementation constraints (such as power consumption or communication of the output to the user), aim at a more reasonable and fair estimate of performance.
3. Reporting: Both deterministic and probabilistic measures of performance should be reported, ideally favoring those most commonly observed in this review.

As such, beyond setting a performance benchmark for future developments in automated forecasting algorithms, we hope that the considerations found in this work drive future efforts into a convergent approach regarding study design and evaluation methodologies, in a way that reflects how the clinical application of forecasting of seizure likelihood is currently envisioned.

#### Limitations

This work identified some limitations in the studies included in the review. The retrospective approach during training and evaluation implies that temporal integrity is not respected, which introduces biases not only when computing profiles with cyclic nature but also when accounting for the natural evolution of patients’ surrogate patterns of the preictal state across time. In this context, prospective evaluation is a valuable step towards the integration of these algorithms into the clinical setting, as it provides a more comprehensive estimate on their capacity to generalize [45]. While only one study [29] provided a prospective period of algorithm evaluation, several have opted for a pseudo-prospective approach.

It is also important to highlight some limitations of this work. Firstly, determining the forecast horizon proved to be somewhat of a challenge. Despite efforts made by Proix et al. [22] to elucidate this concept, the forecast horizon is not consistently defined across studies (only 8 out of the 18 studies explicitly stated it): some studies propose algorithms that generate forecasts at specific clock times, naturally defining the horizon as the time between forecasts; while others solely provide the SPH and/or SOP used during event-based evaluation, without offering a clear conversion into a horizon interpretable by the user. Secondly, only a fraction of the proposed algorithms was included in each meta-analysis, despite the considerable number of algorithms found in the literature. Thirdly, there was significant unexplained heterogeneity in both meta-analyses, which may suggest the discrepancy within study designs as a potential culprit. Moreover, the significant decrease in heterogeneity when subgrouping by study may also suggest the relevance of consistency in study design for the homogeneity and robustness of future meta-analysis. The analysis on moderator effect provided very little explanation on the observed heterogeneity and the multiple subgroup analyses revealed significant imbalance in the number of approaches proposed across the different design variables.

While the results of this work provide a well-defined starting point for future developments and performance benchmarks according to study design, it is important to acknowledge that the heterogeneity here characterized may compromise pooled results and direct comparison between performances. For this reason, readers are advised to interpret the results of the meta-analyses with caution. Instead, readers are encouraged to leverage the insights from this work to identify the gaps in study design that remain underexplored and, once addressed, will enable a more reliable, quantitative summary of the state-of-the-art.

## Supplementary Information

Below is the link to the electronic supplementary material.

Supplementary file 1 (xlsx 16 KB)

Supplementary file 2 (pdf 4481 KB)

## Data Availability

All data used for the characterization of the studies in this review, as well as for the meta-analysis are available as Supplementary Material. The details of the protocol for this systematic review are available on PROSPERO, under PROSPERO ID CRD42023478920.

## Footnotes

1 Rayyan software is available at https://www.rayyan.ai/.

2 Following the recommendation of Fu et al. [26], only the cases in which all subgroups had at least four entries were considered eligible for quantification of moderator effect on heterogeneity.

3 The number of reported seizures does not account for the seizures reported in [27], since they only reported the median number of seizures (143, IQR of 13-1233).

4 Although BS was reported in more studies than BSS, the latter was used as a performance metric in a larger number of proposed approaches.

5 Algorithms were considered eligible for the meta-analysis if both the mean and standard deviation (SD) across patients was reported, and if the sample size comprised more than one patient.
