# Supplementary material for: Automated algorithms for seizure forecast: a systematic review and meta-analysis
Source: J Neurol. 2024 Sep 6;271(10):6573–87. doi: 10.1007/s00415-024-12655-z (PMC11447137; doi:10.1007/s00415-024-12655-z)
Supplement: Supplementary file 2 — (pdf 4481 KB) [file 415_2024_12655_MOESM2_ESM.pdf]

Supplementary Material

Automated Algorithms for Seizure Forecast: A Systematic Review and Meta-Analysis

Ana Sofia Carmo, Mariana Abreu, Maria Fortuna Baptista, Miguel de Oliveira Carvalho, Ana Rita Peralta, Ana Fred, Carla Bentes and Hugo Plácido da Silva

This Supplementary Material provides the forest plots for Brier Skill Score (BSS) and Area Under the ROC Curve (AUC), stratified according to type of input data (Surrogate Measures of the Preictal State vs Cyclic Distribution of Events), source of data (i.e. dataset used), input data (EEG vs heart rate vs seizure times vs other inputs), forecast horizon (<1h, 1h, 24h), train/test approach (retrospective vs prospective/pseudo-prospective), and study (i.e. algorithms proposed by the same study). The dashed line illustrates the estimated overall score, and the diamonds represent either the overall or subgroup summary. Additional information provided includes the horizon of the forecast, the sample size, total number of seizures, and the train-test approach (which was either (pseudo-)prospective, P, or retrospective, R). (*) Identifies the work of Leguia et al. that only reported the median number of seizures (143, interquartile range of 13-1233).

Subgroup analysis: Type of input data

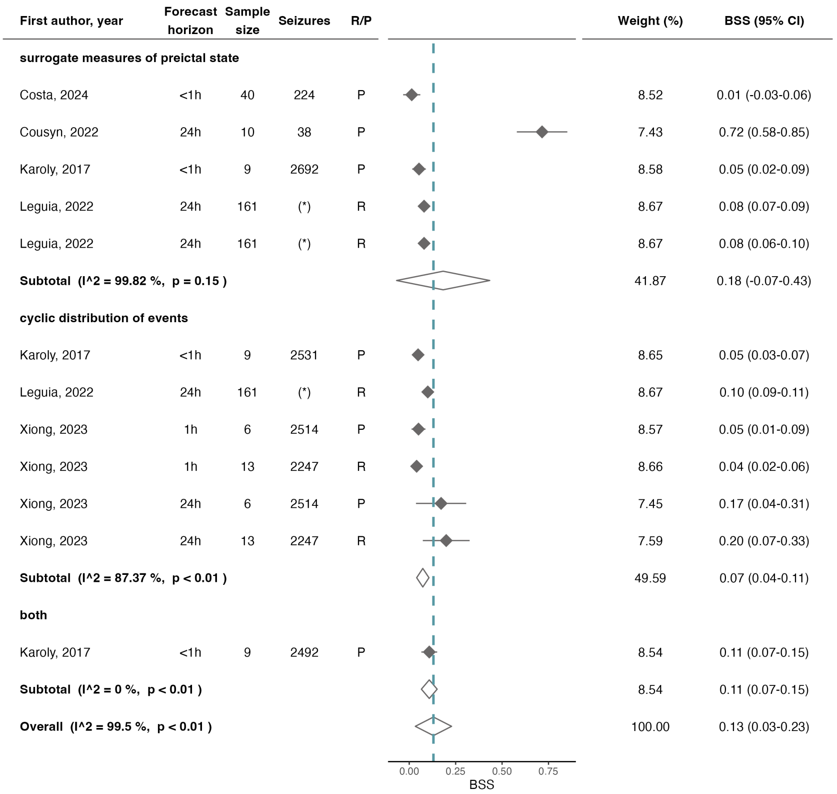

Fig. 1: Forest plot of BSS of seizure forecast algorithms, overall and stratified by type of input data.

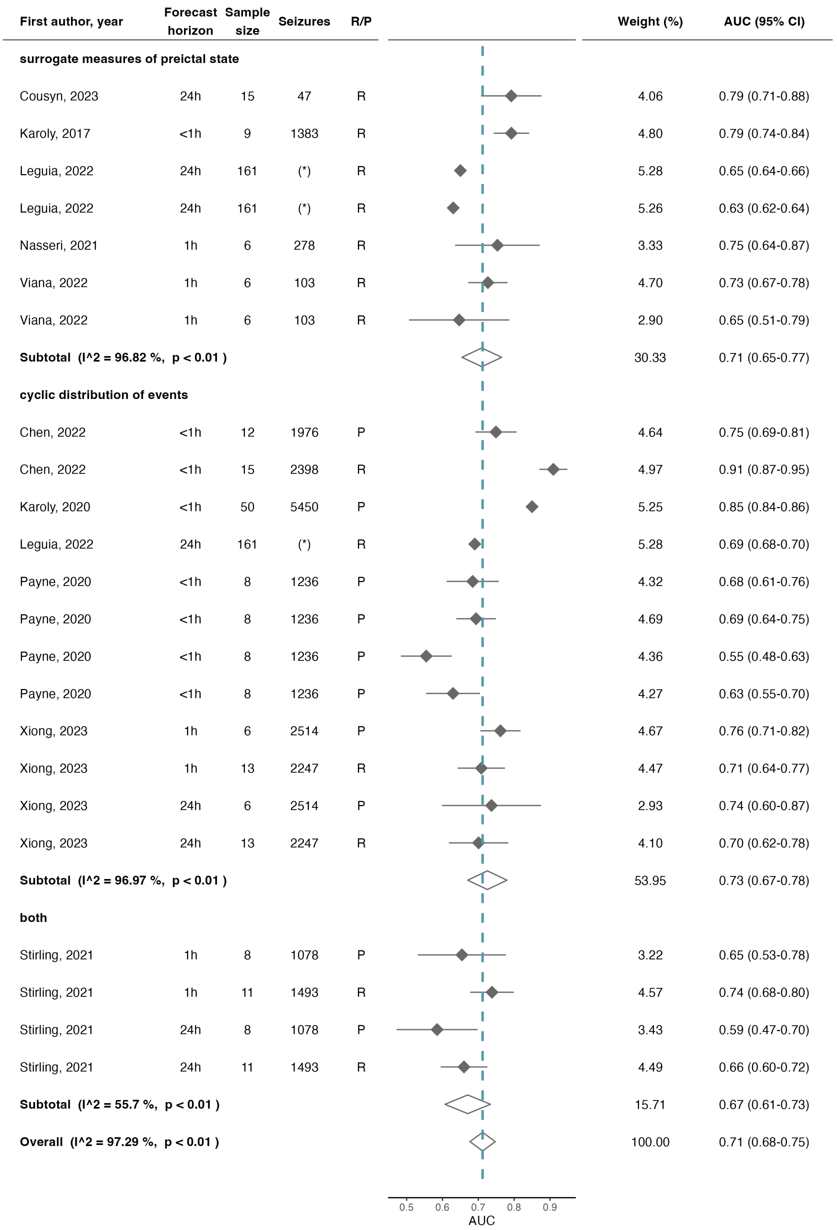

Fig. 2: Forest plot of AUC of seizure forecast algorithms, overall and stratified by type of input data.

Subgroup analysis: Data source

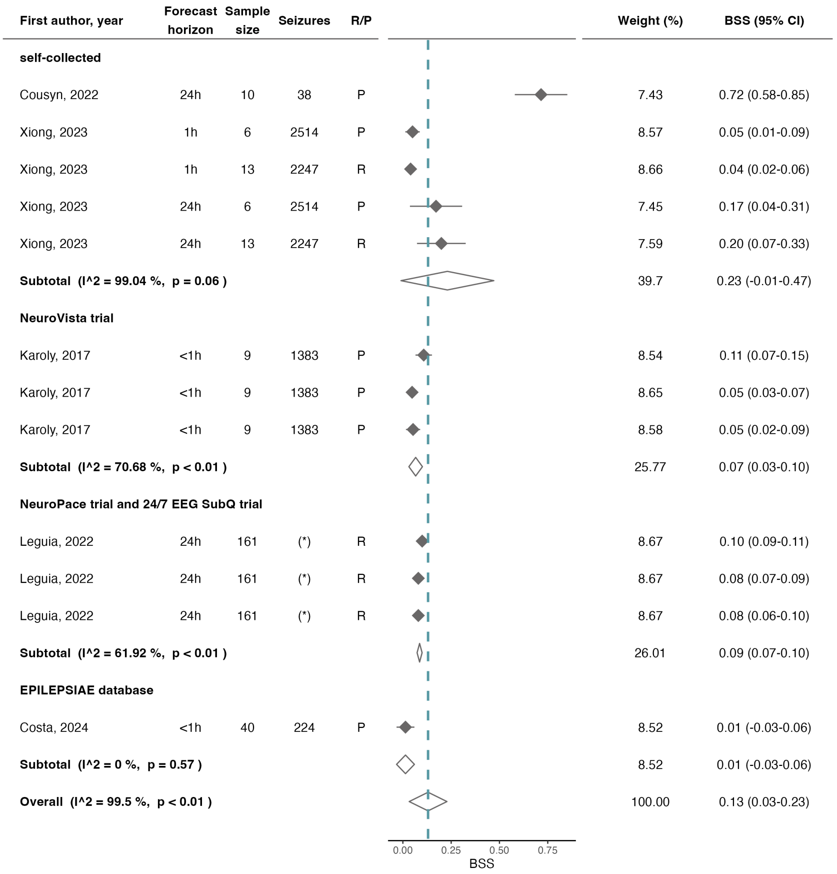

Fig. 3: Forest plot of BSS of seizure forecast algorithms, overall and stratified by data source.

First author, year	Forecast horizon	Sample size	Seizures	R/P	Weight (%)	AUC (95% CI)
**self-collected and NeuroVista trial**						
Karoly, 2020	<1h	50	5450	P	5.25	0.85 (0.84-0.86)
**Subtotal (I^2 = 0 %, p < 0.01)**					5.25	0.85 (0.84-0.86)
**self-collected**						
Cousyn, 2023	24h	15	47	R	4.06	0.79 (0.71-0.88)
Nasseri, 2021	1h	6	278	R	3.33	0.75 (0.64-0.87)
Stirling, 2021	1h	8	1078	P	3.22	0.65 (0.53-0.78)
Stirling, 2021	1h	11	1493	R	4.57	0.74 (0.68-0.80)
Stirling, 2021	24h	8	1078	P	3.43	0.59 (0.47-0.70)
Stirling, 2021	24h	11	1493	R	4.49	0.66 (0.60-0.72)
Xiong, 2023	1h	6	2514	P	4.67	0.76 (0.71-0.82)
Xiong, 2023	1h	13	2247	R	4.47	0.71 (0.64-0.77)
Xiong, 2023	24h	6	2514	P	2.93	0.74 (0.60-0.87)
Xiong, 2023	24h	13	2247	R	4.10	0.70 (0.62-0.78)
**Subtotal (I^2 = 42.3 %, p < 0.01)**					39.27	0.71 (0.68-0.75)
**NeuroVista trial**						
Chen, 2022	<1h	12	1976	P	4.64	0.75 (0.69-0.81)
Chen, 2022	<1h	15	2398	R	4.97	0.91 (0.87-0.95)
Karoly, 2017	<1h	9	1383	R	4.80	0.79 (0.74-0.84)
Payne, 2020	<1h	8	1236	P	4.32	0.68 (0.61-0.76)
Payne, 2020	<1h	8	1236	P	4.69	0.69 (0.64-0.75)
Payne, 2020	<1h	8	1236	P	4.36	0.55 (0.48-0.63)
Payne, 2020	<1h	8	1236	P	4.27	0.63 (0.55-0.70)
**Subtotal (I^2 = 93.71 %, p < 0.01)**					32.05	0.72 (0.63-0.80)
**NeuroPace trial and 24/7 EEG SubQ trial**						
Leguia, 2022	24h	161	(*)	R	5.28	0.69 (0.68-0.70)
Leguia, 2022	24h	161	(*)	R	5.28	0.65 (0.64-0.66)
Leguia, 2022	24h	161	(*)	R	5.26	0.63 (0.62-0.64)
**Subtotal (I^2 = 97.42 %, p < 0.01)**					15.82	0.66 (0.62-0.69)
**24/7 EEG SubQ trial**						
Viana, 2022	1h	6	103	R	4.70	0.73 (0.67-0.78)
Viana, 2022	1h	6	103	R	2.90	0.65 (0.51-0.79)
**Subtotal (I^2 = 8.3 %, p < 0.01)**					7.6	0.71 (0.66-0.77)
**Overall (I^2 = 97.29 %, p < 0.01)**					100.00	0.71 (0.68-0.75)

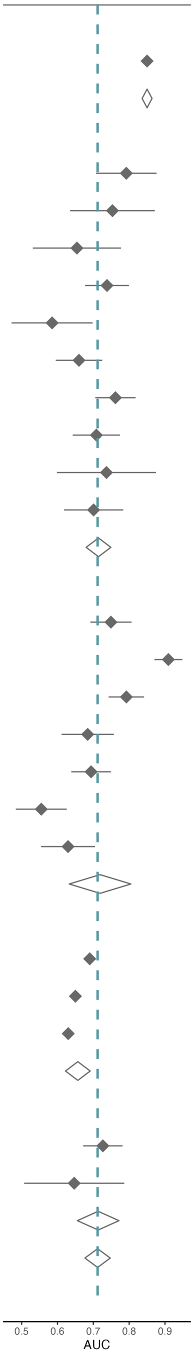

Fig. 4: Forest plot of AUC of seizure forecast algorithms, overall and stratified by data source.

Subgroup analysis: Input data

First author, year	Forecast horizon	Sample size	Seizures	R/P	Weight (%)	BSS (95% CI)
**seizure times and others**						
Xiong, 2023	1h	6	2514	P	8.57	0.05 (0.01-0.09)
Xiong, 2023	1h	13	2247	R	8.66	0.04 (0.02-0.06)
Xiong, 2023	24h	6	2514	P	7.45	0.17 (0.04-0.31)
Xiong, 2023	24h	13	2247	R	7.59	0.20 (0.07-0.33)
**Subtotal (I^2 = 87.56 %, p = 0.01)**					32.27	0.09 (0.02-0.16)
**seizure times**						
Karoly, 2017	<1h	9	1383	P	8.65	0.05 (0.03-0.07)
Leguia, 2022	24h	161	(*)	R	8.67	0.08 (0.06-0.10)
**Subtotal (I^2 = 82.97 %, p < 0.01)**					17.32	0.06 (0.03-0.10)
**EEG and seizure times**						
Karoly, 2017	<1h	9	1383	P	8.54	0.11 (0.07-0.15)
**Subtotal (I^2 = 0 %, p < 0.01)**					8.54	0.11 (0.07-0.15)
**EEG**						
Costa, 2024	<1h	40	224	P	8.52	0.01 (-0.03-0.06)
Cousyn, 2022	24h	10	38	P	7.43	0.72 (0.58-0.85)
Karoly, 2017	<1h	9	1383	P	8.58	0.05 (0.02-0.09)
Leguia, 2022	24h	161	(*)	R	8.67	0.10 (0.09-0.11)
Leguia, 2022	24h	161	(*)	R	8.67	0.08 (0.07-0.09)
**Subtotal (I^2 = 99.82 %, p = 0.14)**					41.87	0.19 (-0.06-0.44)
**Overall (I^2 = 99.5 %, p < 0.01)**					100.00	0.13 (0.03-0.23)

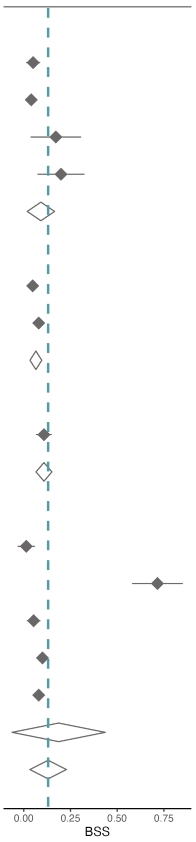

Fig. 5: Forest plot of BSS of seizure forecast algorithms, overall and stratified by input data.

First author, year	Forecast horizon	Sample size	Seizures	R/P	Weight (%)	AUC (95% CI)
**seizure times and others**						
Payne, 2020	<1h	8	1236	P	4.32	0.68 (0.61-0.76)
Stirling, 2021	1h	8	1078	P	3.22	0.65 (0.53-0.78)
Stirling, 2021	1h	11	1493	R	4.57	0.74 (0.68-0.80)
Stirling, 2021	24h	8	1078	P	3.43	0.59 (0.47-0.70)
Stirling, 2021	24h	11	1493	R	4.49	0.66 (0.60-0.72)
Xiong, 2023	1h	6	2514	P	4.67	0.76 (0.71-0.82)
Xiong, 2023	1h	13	2247	R	4.47	0.71 (0.64-0.77)
Xiong, 2023	24h	6	2514	P	2.93	0.74 (0.60-0.87)
Xiong, 2023	24h	13	2247	R	4.10	0.70 (0.62-0.78)
**Subtotal (I^2 = 35.52 %, p < 0.01)**					36.2	0.70 (0.67-0.73)
**seizure times**						
Karoly, 2020	<1h	50	5450	P	5.25	0.85 (0.84-0.86)
Leguia, 2022	24h	161	(*)	R	5.26	0.63 (0.62-0.64)
Payne, 2020	<1h	8	1236	P	4.69	0.69 (0.64-0.75)
**Subtotal (I^2 = 99.37 %, p < 0.01)**					15.2	0.73 (0.59-0.86)
**others**						
Nasseri, 2021	1h	6	278	R	3.33	0.75 (0.64-0.87)
Payne, 2020	<1h	8	1236	P	4.36	0.55 (0.48-0.63)
Payne, 2020	<1h	8	1236	P	4.27	0.63 (0.55-0.70)
**Subtotal (I^2 = 78.64 %, p < 0.01)**					11.96	0.64 (0.53-0.74)
**HR**						
Cousyn, 2023	24h	15	47	R	4.06	0.79 (0.71-0.88)
**Subtotal (I^2 = 0 %, p < 0.01)**					4.06	0.79 (0.71-0.88)
**EEG and others**						
Viana, 2022	1h	6	103	R	4.70	0.73 (0.67-0.78)
**Subtotal (I^2 = 0 %, p < 0.01)**					4.7	0.73 (0.67-0.78)
**EEG**						
Chen, 2022	<1h	12	1976	P	4.64	0.75 (0.69-0.81)
Chen, 2022	<1h	15	2398	R	4.97	0.91 (0.87-0.95)
Karoly, 2017	<1h	9	1383	R	4.80	0.79 (0.74-0.84)
Leguia, 2022	24h	161	(*)	R	5.28	0.69 (0.68-0.70)
Leguia, 2022	24h	161	(*)	R	5.28	0.65 (0.64-0.66)
Viana, 2022	1h	6	103	R	2.90	0.65 (0.51-0.79)
**Subtotal (I^2 = 99.14 %, p < 0.01)**					27.87	0.74 (0.66-0.83)
**Overall (I^2 = 97.29 %, p < 0.01)**					100.00	0.71 (0.68-0.75)

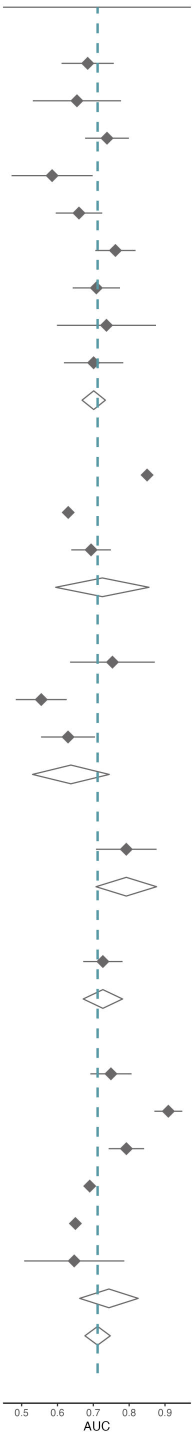

Fig. 6: Forest plot of AUC of seizure forecast algorithms, overall and stratified by input data.

Subgroup analysis: Forecast horizon

First author, year	Forecast horizon	Sample size	Seizures	R/P	Weight (%)	BSS (95% CI)
**24h**						
Cousyn, 2022	24h	10	38	P	7.43	0.72 (0.58-0.85)
Leguia, 2022	24h	161	(*)	R	8.67	0.10 (0.09-0.11)
Leguia, 2022	24h	161	(*)	R	8.67	0.08 (0.07-0.09)
Leguia, 2022	24h	161	(*)	R	8.67	0.08 (0.06-0.10)
Xiong, 2023	24h	6	2514	P	7.45	0.17 (0.04-0.31)
Xiong, 2023	24h	13	2247	R	7.59	0.20 (0.07-0.33)
**Subtotal (I^2 = 99.78 %, p = 0.02)**					48.48	0.22 (0.03-0.41)
**1h**						
Xiong, 2023	1h	6	2514	P	8.57	0.05 (0.01-0.09)
Xiong, 2023	1h	13	2247	R	8.66	0.04 (0.02-0.06)
**Subtotal (I^2 = 0 %, p < 0.01)**					17.23	0.04 (0.03-0.06)
**<1h**						
Costa, 2024	<1h	40	224	P	8.52	0.01 (-0.03-0.06)
Karoly, 2017	<1h	9	1383	P	8.54	0.11 (0.07-0.15)
Karoly, 2017	<1h	9	1383	P	8.65	0.05 (0.03-0.07)
Karoly, 2017	<1h	9	1383	P	8.58	0.05 (0.02-0.09)
**Subtotal (I^2 = 73.74 %, p < 0.01)**					34.29	0.06 (0.02-0.09)
**Overall (I^2 = 99.5 %, p < 0.01)**					100.00	0.13 (0.03-0.23)

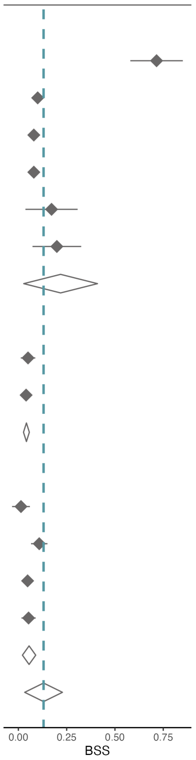

Fig. 7: Forest plot of BSS of seizure forecast algorithms, overall and stratified by forecast horizon.

First author, year	Forecast horizon	Sample size	Seizures	R/P	Weight (%)	AUC (95% CI)
**24h**						
Cousyn, 2023	24h	15	47	R	4.06	0.79 (0.71-0.88)
Leguia, 2022	24h	161	(*)	R	5.28	0.69 (0.68-0.70)
Leguia, 2022	24h	161	(*)	R	5.28	0.65 (0.64-0.66)
Leguia, 2022	24h	161	(*)	R	5.26	0.63 (0.62-0.64)
Stirling, 2021	24h	8	1078	P	3.43	0.59 (0.47-0.70)
Stirling, 2021	24h	11	1493	R	4.49	0.66 (0.60-0.72)
Xiong, 2023	24h	6	2514	P	2.93	0.74 (0.60-0.87)
Xiong, 2023	24h	13	2247	R	4.10	0.70 (0.62-0.78)
**Subtotal (I^2 = 95.25 %, p < 0.01)**					34.83	0.67 (0.64-0.71)
**1h**						
Nasseri, 2021	1h	6	278	R	3.33	0.75 (0.64-0.87)
Stirling, 2021	1h	8	1078	P	3.22	0.65 (0.53-0.78)
Stirling, 2021	1h	11	1493	R	4.57	0.74 (0.68-0.80)
Viana, 2022	1h	6	103	R	4.70	0.73 (0.67-0.78)
Viana, 2022	1h	6	103	R	2.90	0.65 (0.51-0.79)
Xiong, 2023	1h	6	2514	P	4.67	0.76 (0.71-0.82)
Xiong, 2023	1h	13	2247	R	4.47	0.71 (0.64-0.77)
**Subtotal (I^2 = 0 %, p < 0.01)**					27.86	0.73 (0.70-0.76)
**<1h**						
Chen, 2022	<1h	12	1976	P	4.64	0.75 (0.69-0.81)
Chen, 2022	<1h	15	2398	R	4.97	0.91 (0.87-0.95)
Karoly, 2017	<1h	9	1383	R	4.80	0.79 (0.74-0.84)
Karoly, 2020	<1h	50	5450	P	5.25	0.85 (0.84-0.86)
Payne, 2020	<1h	8	1236	P	4.32	0.68 (0.61-0.76)
Payne, 2020	<1h	8	1236	P	4.69	0.69 (0.64-0.75)
Payne, 2020	<1h	8	1236	P	4.36	0.55 (0.48-0.63)
Payne, 2020	<1h	8	1236	P	4.27	0.63 (0.55-0.70)
**Subtotal (I^2 = 96.31 %, p < 0.01)**					37.3	0.74 (0.66-0.82)
**Overall (I^2 = 97.29 %, p < 0.01)**					100.00	0.71 (0.68-0.75)

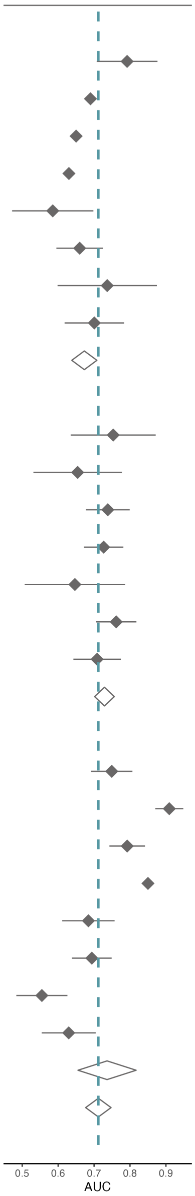

Fig. 8: Forest plot of AUC of seizure forecast algorithms, overall and stratified by forecast horizon.

Subgroup analysis: Train-test approach

First author, year	Forecast horizon	Sample size	Seizures	R/P	Weight (%)	BSS (95% CI)
**retrospective**						
Leguia, 2022	24h	161	(*)	R	8.67	0.10 (0.09-0.11)
Leguia, 2022	24h	161	(*)	R	8.67	0.08 (0.07-0.09)
Leguia, 2022	24h	161	(*)	R	8.67	0.08 (0.06-0.10)
Xiong, 2023	1h	13	2247	R	8.66	0.04 (0.02-0.06)
Xiong, 2023	24h	13	2247	R	7.59	0.20 (0.07-0.33)
**Subtotal (I^2 = 89.98 %, p < 0.01)**					42.26	0.08 (0.05-0.11)
**prospective**						
Costa, 2024	<1h	40	224	P	8.52	0.01 (-0.03-0.06)
Cousyn, 2022	24h	10	38	P	7.43	0.72 (0.58-0.85)
Karoly, 2017	<1h	9	1383	P	8.54	0.11 (0.07-0.15)
Karoly, 2017	<1h	9	1383	P	8.65	0.05 (0.03-0.07)
Karoly, 2017	<1h	9	1383	P	8.58	0.05 (0.02-0.09)
Xiong, 2023	1h	6	2514	P	8.57	0.05 (0.01-0.09)
Xiong, 2023	24h	6	2514	P	7.45	0.17 (0.04-0.31)
**Subtotal (I^2 = 99.18 %, p = 0.08)**					57.74	0.16 (-0.02-0.34)
**Overall (I^2 = 99.5 %, p < 0.01)**					100.00	0.13 (0.03-0.23)

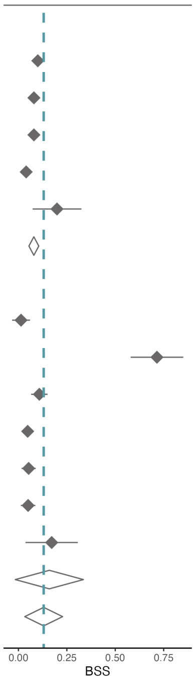

Fig. 9: Forest plot of BSS of seizure forecast algorithms, overall and stratified by train-test approach.

First author, year	Forecast horizon	Sample size	Seizures	R/P	Weight (%)	AUC (95% CI)
**retrospective**						
Chen, 2022	<1h	15	2398	R	4.97	0.91 (0.87-0.95)
Cousyn, 2023	24h	15	47	R	4.06	0.79 (0.71-0.88)
Karoly, 2017	<1h	9	1383	R	4.80	0.79 (0.74-0.84)
Leguia, 2022	24h	161	(*)	R	5.28	0.69 (0.68-0.70)
Leguia, 2022	24h	161	(*)	R	5.28	0.65 (0.64-0.66)
Leguia, 2022	24h	161	(*)	R	5.26	0.63 (0.62-0.64)
Nasseri, 2021	1h	6	278	R	3.33	0.75 (0.64-0.87)
Stirling, 2021	1h	11	1493	R	4.57	0.74 (0.68-0.80)
Stirling, 2021	24h	11	1493	R	4.49	0.66 (0.60-0.72)
Viana, 2022	1h	6	103	R	4.70	0.73 (0.67-0.78)
Viana, 2022	1h	6	103	R	2.90	0.65 (0.51-0.79)
Xiong, 2023	1h	13	2247	R	4.47	0.71 (0.64-0.77)
Xiong, 2023	24h	13	2247	R	4.10	0.70 (0.62-0.78)
**Subtotal (I^2 = 97.88 %, p < 0.01)**					58.21	0.72 (0.68-0.77)
**prospective**						
Chen, 2022	<1h	12	1976	P	4.64	0.75 (0.69-0.81)
Karoly, 2020	<1h	50	5450	P	5.25	0.85 (0.84-0.86)
Payne, 2020	<1h	8	1236	P	4.32	0.68 (0.61-0.76)
Payne, 2020	<1h	8	1236	P	4.69	0.69 (0.64-0.75)
Payne, 2020	<1h	8	1236	P	4.36	0.55 (0.48-0.63)
Payne, 2020	<1h	8	1236	P	4.27	0.63 (0.55-0.70)
Stirling, 2021	1h	8	1078	P	3.22	0.65 (0.53-0.78)
Stirling, 2021	24h	8	1078	P	3.43	0.59 (0.47-0.70)
Xiong, 2023	1h	6	2514	P	4.67	0.76 (0.71-0.82)
Xiong, 2023	24h	6	2514	P	2.93	0.74 (0.60-0.87)
**Subtotal (I^2 = 90.22 %, p < 0.01)**					41.78	0.70 (0.64-0.75)
**Overall (I^2 = 97.29 %, p < 0.01)**					100.00	0.71 (0.68-0.75)

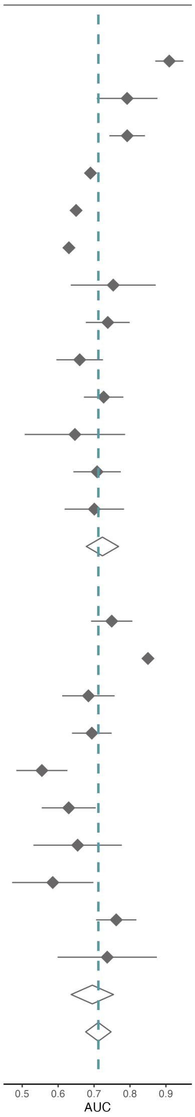

Fig. 10: Forest plot of AUC of seizure forecast algorithms, overall and stratified by train-test approach.

Subgroup analysis: Study

First author, year	Forecast horizon	Sample size	Seizures	R/P	Weight (%)	BSS (95% CI)
**Xiong, 2023**						
	1h	6	2514	P	8.57	0.05 (0.01-0.09)
	1h	13	2247	R	8.66	0.04 (0.02-0.06)
	24h	6	2514	P	7.45	0.17 (0.04-0.31)
	24h	13	2247	R	7.59	0.20 (0.07-0.33)
**Subtotal (I^2 = 87.56 %, p = 0.01)**					32.27	0.09 (0.02-0.16)
**Leguia, 2022**						
	24h	161	(*)	R	8.67	0.10 (0.09-0.11)
	24h	161	(*)	R	8.67	0.08 (0.07-0.09)
	24h	161	(*)	R	8.67	0.08 (0.06-0.10)
**Subtotal (I^2 = 61.92 %, p < 0.01)**					26.01	0.09 (0.07-0.10)
**Karoly, 2017**						
	<1h	9	1383	P	8.54	0.11 (0.07-0.15)
	<1h	9	1383	P	8.65	0.05 (0.03-0.07)
	<1h	9	1383	P	8.58	0.05 (0.02-0.09)
**Subtotal (I^2 = 70.68 %, p < 0.01)**					25.77	0.07 (0.03-0.10)
**Cousyn, 2022**						
	24h	10	38	P	7.43	0.72 (0.58-0.85)
**Subtotal (I^2 = 0 %, p < 0.01)**					7.43	0.72 (0.58-0.85)
**Costa, 2024**						
	<1h	40	224	P	8.52	0.01 (-0.03-0.06)
**Subtotal (I^2 = 0 %, p = 0.57)**					8.52	0.01 (-0.03-0.06)
**Overall (I^2 = 99.5 %, p < 0.01)**					100.00	0.13 (0.03-0.23)

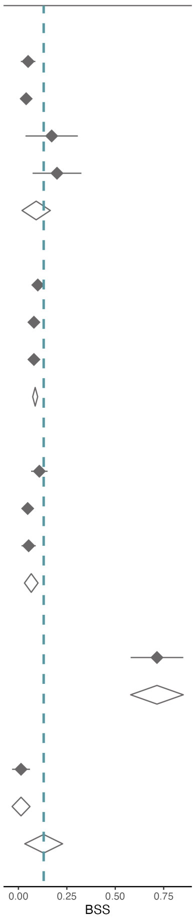

Fig. 11: Forest plot of BSS of seizure forecast algorithms, overall and stratified by study.

First author, year	Forecast horizon	Sample size	Seizures	R/P	Weight (%)	AUC (95% CI)
**Xiong, 2023**						
	1h	6	2514	P	4.67	0.76 (0.71-0.82)
	1h	13	2247	R	4.47	0.71 (0.64-0.77)
	24h	6	2514	P	2.93	0.74 (0.60-0.87)
	24h	13	2247	R	4.10	0.70 (0.62-0.78)
**Subtotal (I^2 = 2.75 %, p < 0.01)**					16.17	0.73 (0.69-0.77)
**Viana, 2022**						
	1h	6	103	R	4.70	0.73 (0.67-0.78)
	1h	6	103	R	2.90	0.65 (0.51-0.79)
**Subtotal (I^2 = 8.3 %, p < 0.01)**					7.6	0.71 (0.66-0.77)
**Stirling, 2021**						
	1h	8	1078	P	3.22	0.65 (0.53-0.78)
	1h	11	1493	R	4.57	0.74 (0.68-0.80)
	24h	8	1078	P	3.43	0.59 (0.47-0.70)
	24h	11	1493	R	4.49	0.66 (0.60-0.72)
**Subtotal (I^2 = 55.7 %, p < 0.01)**					15.71	0.67 (0.61-0.73)
**Payne, 2020**						
	<1h	8	1236	P	4.32	0.68 (0.61-0.76)
	<1h	8	1236	P	4.69	0.69 (0.64-0.75)
	<1h	8	1236	P	4.36	0.55 (0.48-0.63)
	<1h	8	1236	P	4.27	0.63 (0.55-0.70)
**Subtotal (I^2 = 70.86 %, p < 0.01)**					17.64	0.64 (0.58-0.71)
**Nasseri, 2021**						
	1h	6	278	R	3.33	0.75 (0.64-0.87)
**Subtotal (I^2 = 0 %, p < 0.01)**					3.33	0.75 (0.64-0.87)
**Leguia, 2022**						
	24h	161	(*)	R	5.28	0.69 (0.68-0.70)
	24h	161	(*)	R	5.28	0.65 (0.64-0.66)
	24h	161	(*)	R	5.26	0.63 (0.62-0.64)
**Subtotal (I^2 = 97.42 %, p < 0.01)**					15.82	0.66 (0.62-0.69)
**Karoly, 2020**						
	<1h	50	5450	P	5.25	0.85 (0.84-0.86)
**Subtotal (I^2 = 0 %, p < 0.01)**					5.25	0.85 (0.84-0.86)
**Karoly, 2017**						
	<1h	9	1383	R	4.80	0.79 (0.74-0.84)
**Subtotal (I^2 = 0 %, p < 0.01)**					4.8	0.79 (0.74-0.84)
**Cousyn, 2023**						
	24h	15	47	R	4.06	0.79 (0.71-0.88)
**Subtotal (I^2 = 0 %, p < 0.01)**					4.06	0.79 (0.71-0.88)
**Chen, 2022**						
	<1h	12	1976	P	4.64	0.75 (0.69-0.81)
	<1h	15	2398	R	4.97	0.91 (0.87-0.95)
**Subtotal (I^2 = 95.1 %, p < 0.01)**					9.61	0.83 (0.67-0.99)
**Overall (I^2 = 97.29 %, p < 0.01)**					100.00	0.71 (0.68-0.75)

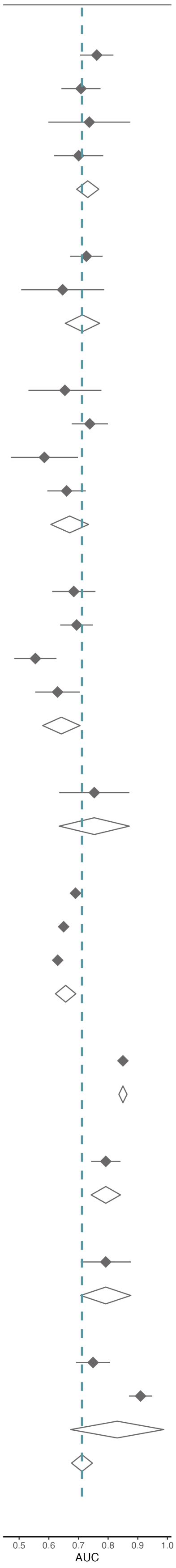

Fig. 12: Forest plot of AUC of seizure forecast algorithms, overall and stratified by study.
